# Self-rated health and reasons for non-vaccination against seasonal influenza in Canadian adults with asthma

**DOI:** 10.1371/journal.pone.0172117

**Published:** 2017-02-16

**Authors:** Jennifer L. Guthrie, David Fisman, Jennifer L. Gardy

**Affiliations:** 1 School of Population and Public Health, University of British Columbia, Vancouver, British Columbia, Canada; 2 Dalla Lana School of Public Health, University of Toronto, Toronto, Ontario, Canada; New York City Department of Health and Mental Hygiene, UNITED STATES

## Abstract

**Introduction:**

While seasonal influenza vaccination is recommended for individuals with asthma, uptake in this population is low. We examined how self-rated health impacts reasons for not being immunized against influenza in Canadian adults with asthma, focusing on those who have never been immunized.

**Methods:**

We pooled four cycles of the Canadian Community Health Survey (cycles 3.1(2005), 2007/08, 2009/10 and 2011/12), grouping individuals by whether their reasons for not having been vaccinated were perceptual or technical. We used a multivariable logistic regression model, adjusted for confounders, to quantify the relationship between self-rated health and their reported reasons for not vaccinating.

**Results:**

Among the 9,836 respondents, 84.4% cited perceptual barriers as a reason for not being vaccinated. After adjusting for socio-demographic characteristics and province of residence, we determined that reporting perceptual barriers was associated with self-rated health status, with the adjusted odds ratios ranging from 1.42 (95%CI: 0.97, 2.09) to 2.64 (95%CI: 1.74, 3.99) for fair and excellent health versus poor health, respectively. Each increase in self-rated health category was associated with greater odds of citing a perceptual rather than technical barrier as a reason for non-vaccination.

**Discussion:**

Self-reported health influences people’s perception of the need for influenza vaccination. Viewing the results through the lens of the precaution adoption process model suggests that personalizing communication around both the risk of influenza and the effectiveness of the vaccine may improve uptake amongst adults with asthma.

## Introduction

Influenza is a significant public health concern—an estimated 10 to 20% of the population is infected each year, and though many recover from acute symptoms within a few days, some develop serious illness requiring hospitalization [1,2]. In Canada, influenza is estimated to cause more than 12,000 hospitalizations and 3,500 deaths annually [3]. Severe illness is most common in the very young and elderly as well as those with chronic conditions, particularly those with underlying respiratory conditions, including asthma [4]. Indeed, asthma is the most common chronic condition observed in both adult and paediatric patients hospitalized with influenza [4,5].

According to Statistics Canada, 2.2 million Canadian adults are living with asthma [6], representing a large group of individuals at risk for influenza-related complications. Vaccination remains the best preventive measure available and is offered at no charge across Canada to individuals considered at high risk for complications [7]; however, reports from North America and Europe suggest that uptake of influenza vaccination amongst individuals with asthma is low, ranging from 30 to 50%, and uptake in this group is lower than amongst individuals with other chronic conditions [8].

Previous work has shown that individuals who perceive themselves as “healthy” are less likely to participate in certain types of preventive health activities, such as blood pressure checks, cholesterol testing, and influenza immunization—a phenomenon that holds true even amongst those with chronic conditions [9,10]. Recently, Nowak et al. [11] found that individuals self-reporting good health were less likely to see a need for influenza vaccination, and some with chronic conditions felt that managing their disease well negated the need for seasonal influenza vaccination. Similarly, researchers have noted that self-rated health is one of the reasons for poor uptake of annual influenza vaccination in individuals with asthma, where the likelihood of vaccination decreases as self-rated health status improves [12].

Here we explore how self-rated health impacts attitudes towards influenza vaccination in Canadian adults with asthma, for whom seasonal influenza vaccination is highly recommended in all provinces and territories. Using Canadian Community Health Survey data, we focus specifically on asthmatic adults who self-reported having never been immunized against influenza. We group respondents into those who were not vaccinated because they perceived it as unnecessary and those whose reasons for not vaccinating reflect technical barriers, such as access to health care or fear of vaccination. While previous work has examined influenza vaccine uptake in defined age groups and in populations with particular chronic diseases [11,13–15], no studies to date have focused specifically on Canadian adults with asthma and their reasons for non-vaccination, specifically perceived versus technical barriers to immunization.

## Methods

### Study dataset

The Canadian Community Health Survey (CCHS) is a national population-based survey administered by Statistics Canada that collects self-reported information on health status, health service utilization, and determinants of population health. The CCHS uses a multistage, stratified cluster design to target the household population aged 12 and older in all provinces and territories, with approximately 98% of the Canadian population targeted for inclusion. The remaining 2% not represented includes full-time members of the Canadian Forces, as well as residents of institutions, reserves, and some remote areas. The analyses presented here used Statistics Canada’s Public Use Microdata Files (PUMFs) [16] to create a combined dataset spanning eight years (2005–2012) of the CCHS (*n* = 512,399). Prior to 2007, data collection occurred every two years, after which surveys were conducted annually with data released as a two-year cycle. Cycles 3.1(2005), 2007/08, 2009/10, and 2011/12 were administered from January through December of the indicated cycle years, and have an average sample population of 128,100 participants, with person-level response rates ranging from 87.3% (2011/12) to 92.9% (2005). We combined cycles to account for variation in vaccine uptake during and after pandemic influenza and respiratory outbreak years [17,18] using the pooled approach described by Thomas and Wannell [19]. Further details of CCHS sampling and interviewing methods are available from Statistics Canada [20].

### Analytic sample

**Fig 1:**
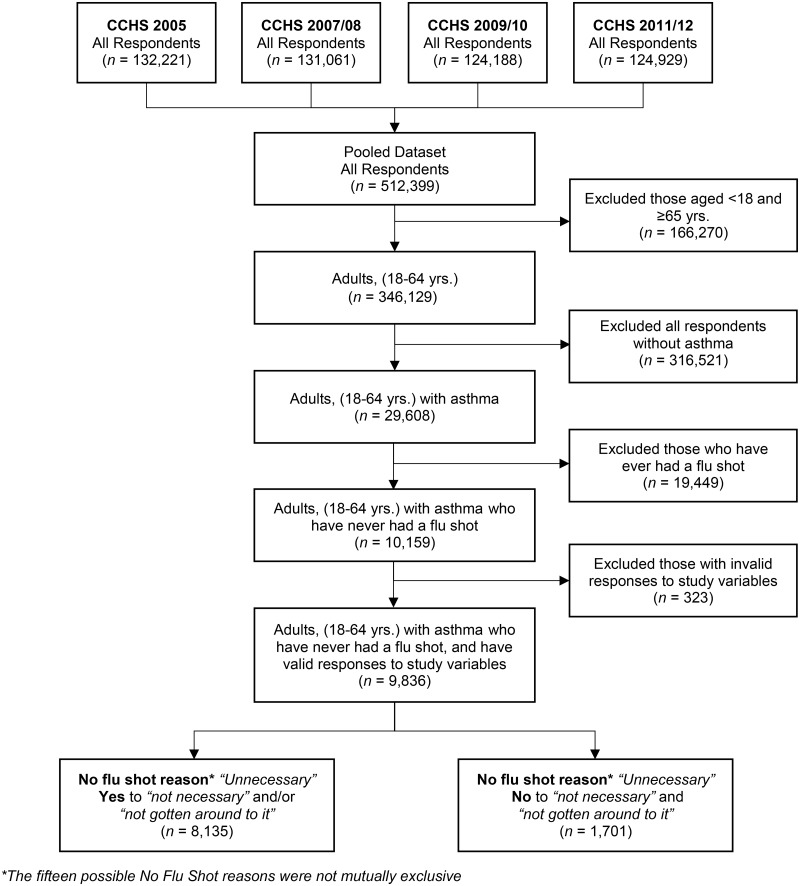
Analytic sample. Selection of the final analytic sample, examining the association between self-reported health and the reasons for non-vaccination against seasonal influenza among Canadian adults with asthma, using pooled CCHS cycles 3.1(2005), 2007/08, 2009/10, 2011/12. Invalid responses included *don't know*, *refusal*, and *not stated*.

The sample consisted of respondents with a self-reported diagnosis of asthma and was limited to adults between the ages of 18 and 64 –a population in which vaccination decisions are more likely to be at the discretion of the individual and not influenced by health care providers, caregivers or parents/guardians. We further limited our analysis to include only respondents indicating that they have never been vaccinated against seasonal influenza. Those with invalid responses (*don’t know*, *refusal* or *not stated*) for any of the study variables, including confounders, were excluded. The final analytic sample consisted of 9,836 respondents (Fig 1).

### Study variables

With respect to seasonal influenza vaccination, the CCHS asks, “Have you ever had a seasonal flu shot?” Amongst those who responded that they have never been vaccinated against seasonal influenza, the survey then asks about reasons for not vaccinating, giving respondents 15 possible reasons. We identified two responses–*“have not gotten around to it”* and *“did not think it was necessary”*–which we together interpreted as perceptual barriers to vaccination, in other words, respondents perceived seasonal influenza vaccination as “*Unnecessary*”. We reasoned that asthmatic individuals reporting never having had a flu shot are consistently remaining unvaccinated despite public health recommendations to the contrary, and thus stating their reason as *“have not gotten around to it”* speaks to their perception of how necessary they feel it is. The other 13 responses were grouped into the category of technical barriers to vaccination, giving a dichotomous outcome variable of individuals reporting either perception- or technical-related barriers to vaccine uptake. Because CCHS permits multiple responses to this survey question, we assigned respondents to the perceptual barrier group if they responded *“have not gotten around to it”* and/or *“did not think it was necessary”*, even if they also reported one or more of the technical barrier responses. Those in the technical barrier group responded No to both *“have not gotten around to it”* and *“did not think it was necessary”* and Yes to any of the other 13 possible responses.

Self-rated health has been demonstrated to be a valid indicator of health status and deemed appropriate for use in general health surveys [21], thus our primary explanatory variable of self-rated health was measured by the survey question, “In general, how would you say your health is now?”. This was analyzed as an ordinal variable with five possible responses (poor, fair, good, very good, or excellent).

Potential confounders identified *a priori* as risk factors for non-vaccination and associated with self-rated health included age, sex, province or territory of residence, and socioeconomic status—assessed through education level (less than secondary school, secondary school graduate, or some level of post-secondary education).

### Analysis plan

Descriptive statistics were calculated for the total survey sample and by age, sex, province/territory, and educational background. Respondents excluded from the study due to invalid responses were compared to those remaining in the analytic sample using chi-square tests to assess potential bias due to missing data. A multivariable logistic regression model was constructed to investigate the relationship between self-rated health and reasons for never having been immunized against influenza.

The pooled dataset was analyzed using SAS, University Edition (SAS Institute Inc., Cary, NC). Probability sampling weights provided by Statistics Canada were applied to all analyses, in a manner appropriate for a pooled dataset [19], to account for the unequal probabilities of selection and nonresponse. Ethics approval for this study was covered by item 7.10.3 of the University of British Columbia's Policy No. 89: Research Involving Human Participants, a clause governing the use of publicly available data sets [22].

## Results

Of 346,129 CCHS respondents aged 18 to 64 years, the (weighted) prevalence of asthma was 8.0%–a rate comparable to estimated asthma prevalence in the United States [23]. Amongst these adults with asthma (29,608), 36.3% reported never having had a flu shot, giving a pool of 10,159 eligible participants. Due to invalid responses to study variables, 323 respondents were excluded, resulting in a final analytic study sample of 9,836 respondents (Fig 1). Compared with respondents who remained in the analytical sample, those who were excluded were more likely to be male (*p* = 0.031), and have a self-rated health status of very good or poor (*p* = 0.033). The two groups did not differ significantly in age, education, or in their distributions across provinces/territories.

Table 1 shows the demographics of the overall study sample, as well as the two groups defined by our outcome variable—those adults with asthma who were unvaccinated because of perceptual barriers and those who were unvaccinated for one or more of the other, technical, reasons given in Table 2. The overall study sample had a higher proportion of females than males– 57.1% and 42.9%, respectively—and across the outcome variable was equally distributed across age groups, with the exception of respondents in the oldest age category (55 to 64 years) who were slightly under-represented (11.1%). The majority of respondents (69.6%) had at least some post-secondary education.

**Table 1:** Characteristics of Canadian Community Health Survey pooled CCHS cycles 3.1(2005), 2007/08, 2009/10, 2011/12, examining the association between self-reported health and the reasons for non-vaccination against seasonal influenza among Canadian adults with asthma.

	Overall Study Sample		Study Sample by No Flu Shot Reason: *“Unnecessary”*	
			%* Yes	%* No
	*n* = 9,836	%*	*n* = 8,135 (84.4%)	*n* = 1,701 (15.6%)
**Self-rated Health**				
Poor	469	3.6	71.2	28.8
Fair	1,187	10.5	78.8	21.2
Good	3,256	33.2	82.2	17.8
Very Good	3,510	36.3	87.7	12.3
Excellent	1,414	16.5	87.9	12.1
**Sex**				
Female	5,869	57.1	81.3	18.7
Male	3,967	42.9	88.6	11.4
**Age**				
18–24 years	1848	21.5	85.1	14.9
25–34 years	2546	26.2	85.5	14.5
35–44 years	2109	21.1	84.4	15.6
45–54 years	1851	20.1	85.7	14.3
55–64 years	1482	11.1	78.1	21.9
**Province/Territory**				
Newfoundland/Labrador	350	1.7	81.0	19.0
Prince Edward Island	166	0.5	84.7	15.3
Nova Scotia	288	2.1	79.4	20.6
New Brunswick	420	2.3	83.3	16.7
Quebec	2525	31.4	86.0	14.0
Ontario	2714	32.1	82.2	17.8
Manitoba	582	4.0	86.0	14.0
Saskatchewan	597	3.0	86.8	13.2
Alberta	908	10.5	89.1	10.9
British Columbia	1091	12.2	82.6	17.4
Yukon/Northwest/Nunavut	195	0.2	80.7	19.3
**Education (individual)**				
Less than secondary	1,649	13.5	82.7	17.3
Secondary	1,737	16.9	84.8	15.2
Post-secondary	6,450	69.6	84.6	15.4

*Percentages are weighted to the Canadian population to account for CCHS multistage stratified sampling strategy

**Table 2:** Reasons for non-vaccination in adults (aged 18–64) with asthma who have never had a flu shot (*n* = 9,836).

	Number	Frequency (%)*
**Reason**†		
Respondent did not think it was necessary	7,214	74.8
Have not gotten around to it	1,116	11.7
Fear	717	7.1
Doctor did not think it was necessary	326	2.8
Bad reaction to previous shot	314	2.7
Did not know where to go	75	1.0
Cost	67	0.7
Not available when required	43	0.6
Personal or family responsibilities	41	0.3
Waiting time was too long	28	0.3
Unable to leave house because of health problem	24	0.3
Not available in area	18	0.1
Transportation problems	8	0.0
Language problems	0	0.0
Other	502	4.2

*Percentages are weighted to the Canadian population to account for CCHS multistage stratified sampling strategy

^†^ Not mutually exclusive, participants could select more than one reason

Overall, 74.8% adults with asthma who had not been vaccinated against seasonal influenza chose *“not necessary”* as a reason for non-vaccination, and 11.7% selected “*not gotten around to it*”, giving a total of 84.4% of respondents reporting a perceptual barrier to vaccination (Tables 1 and 2). With respect to potential confounders, the frequency of perceptual barriers as a reason for non-vaccination was higher in males, respondents under 55 years, those with secondary school education, and residents of the province of Alberta. Amongst our study population, 85.9% of individuals rated their overall health as good, very good or excellent, and those reporting a health status of excellent or very good were most likely to report a perception that influenza vaccination was not necessary (87.9% and 87.7%, respectively) (Table 1).

We then used multivariable logistic regression to examine the relationship between self-rated health status and reasons for non-vaccination against seasonal influenza. In the unadjusted regression model (Table 3), the odds of reporting perceptual barriers to vaccination was associated with higher improved self-rated health in a stepwise manner—the odds increased with each increase in self-rated health status. The association ranged from an odds ratio of 1.50 (95%CI: 1.02, 2.21) for fair versus poor self-rated health to 2.93 (95%CI: 1.96, 4.39) for excellent health versus poor health. After adjusting for confounding, the multivariable model (Table 3) revealed a comparable, although slightly attenuated, result in which the odds of perceiving vaccination as unnecessary ranged from 1.42 (95%CI: 0.97, 2.09) to 2.64 (95%CI: 1.74, 3.99) for fair through excellent health versus poor health, respectively.

**Table 3:** Logistic regression analyses of the relationship between self-rated health and the reasons for non-vaccination (*Unnecessary*–Yes *vs*. No), against seasonal influenza among Canadian adults with asthma, Canadian Community Health Survey pooled cycles 3.1(2005), 2007/08, 2009/10, 2011/12.

	Unadjusted OR (95% CI)	Adjusted OR (95% CI)
**Self-rated Health**		
Poor	Reference	Reference
Fair	1.50 (1.02, 2.21)	1.42 (0.97, 2.09)
Good	1.87 (1.34, 2.62)	1.67 (1.19, 2.34)
Very Good	2.89 (2.06, 4.06)	2.60 (1.84, 3.67)
Excellent	2.93 (1.96, 4.39)	2.64 (1.74, 3.99)
**Sex**		
Female	Reference	Reference
Male	1.79 (1.46, 2.16)	1.78 (1.47, 2.15)
**Age**		
18–24 years	Reference	Reference
25–34 years	1.03 (0.79, 1.35)	1.06 (0.80, 1.41)
35–44 years	0.95 (0.72, 1.25)	1.00 (0.76, 1.32)
45–54 years	1.05 (0.79, 1.39)	1.16 (0.87, 1.56)
55–64 years	0.62 (0.47, 0.83)	0.73 (0.54, 0.98)
**Province/Territory**		
Newfoundland/Labrador	0.93 (0.61, 1.42)	0.92 (0.59, 1.43)
Prince Edward Island	1.20 (0.65, 2.22)	1.24 (0.66, 2.34)
Nova Scotia	0.84 (0.57, 1.23)	0.88 (0.59, 1.31)
New Brunswick	1.09 (0.74, 1.60)	1.15 (0.78, 1.70)
Quebec	1.33 (1.06, 1.68)	1.34 (1.06, 1.69)
Ontario	Reference	Reference
Manitoba	1.33 (0.92, 1.92)	1.31 (0.90, 1.92)
Saskatchewan	1.43 (1.02, 2.01)	1.36 (0.97, 1.90)
Alberta	1.77 (1.28, 2.45)	1.68 (1.22, 2.32)
British Columbia	1.03 (0.77, 1.38)	1.03 (0.76, 1.38)
Yukon/Northwest/Nunavut	0.91 (0.48, 1.71)	0.95 (0.51, 1.77)
**Education (individual)**		
Less than secondary	Reference	Reference
Secondary graduate	1.16 (0.87, 1.56)	1.00 (0.74, 1.36)
Post-secondary	1.15 (0.91, 1.44)	0.95 (0.74, 1.22)

**Abbreviations:** OR—Odds ratio, CI—Confidence interval

Examining the confounders revealed that living in a province outside of Ontario (except Newfoundland, Nova Scotia and the territories) was positively associated with perceiving seasonal influenza vaccination as unnecessary. This may be due in part to Ontario’s early (July, 2000) introduction of a universal influenza vaccination program (vaccine is offered free to all residents), compared to other provinces that did not adopt this approach until after pandemic (H1N1) influenza in 2009, or those who have yet to implement a universal program [7]. Respondents aged 55 to 64, and those with higher education were less likely to report perceptual barriers as a reason for not being vaccinated.

Because the CCHS permits respondents to select multiple responses to the question regarding non-vaccination, we also investigated how these multiple responses might affect our conclusions. We created a third outcome variable–*“both”*–to capture the 3.5% of respondents who answered Yes to *“not necessary”* or *“not gotten around to it”* as well as Yes to any of the other thirteen, more technical reasons. The main effect estimates resulting from this multinomial model were virtually identical to those from the binomial logistic regression, varying by 0.02 points or less (see S1 Table in supplement).

## Discussion

### Summary of main findings

Individuals with asthma, even those with mild asthma and those who are managing their symptoms well with medication, are at increased risk of influenza-related complications [24]. Despite public health efforts to target these and other high-risk individuals for seasonal influenza vaccination, approximately one-third of Canadian adults with asthma have never been vaccinated against influenza. Using Canadian Community Health Survey data, we undertook the first representative, population-based study of adults with asthma in Canada to explore their reasons for non-vaccination.

We found that amongst adults with asthma who had never been vaccinated against influenza, self-rated health status is strongly associated with reporting a perceptual barrier to vaccination, even when controlling for age, sex, province/territory, and education level, and that this relationship shows a stepwise trend, with increasing odds of perceiving vaccination as unnecessary with each successive increase in the level of self-rated health. This suggests that the healthier one perceives themselves, the more likely they are to believe that they do not need to be vaccinated, a trend also observed in research from the United States Behavioral Risk Factor Surveillance System involving adults with asthma who had not been vaccinated in the previous influenza season [12]. Our findings align with those of a previous study exploring influenza vaccination amongst household contacts of young children, and of children with cystic fibrosis—both high-risk groups for whom vaccination of household contacts is recommended. Household contacts cited being “too healthy” as a primary reason for not vaccinating [25]. Similarly a meta-analysis of qualitative studies on influenza vaccination knowledge, attitudes, and beliefs indicated that some individuals with chronic conditions believe seasonal influenza vaccination isn’t needed if their condition is well-managed, with some unaware that their condition placed them at higher risk for complications due to influenza [11]. Amongst the general population, those who perceive themselves to be in good health often expressed the sentiment that seasonal influenza vaccination is either “*not necessary*” or “*optional”* [11].

### Strengths and limitations of the study

The CCHS’ national sampling methodology results in a large survey dataset representative of the Canadian population and is the main strength of this analysis, limiting bias and providing external validity. Furthermore, the confounders—all of which were self-reported—demonstrated associations in the direction expected, adding face validity to the relationship between self-reported health and perceptual barriers to vaccination. However, there are several limitations that should be considered, including the important point that the CCHS is a broad survey and is not designed to specifically examine attitudes or beliefs towards influenza vaccination.

First, the reliability of self-reported influenza vaccination status has not been well-described. Two studies have indicated that it is a highly sensitive and moderately specific measure for flu vaccination status in elderly patients [26,27]. In our study, which included working-age adults, we noted several instances of misclassification, with some respondents (2.7%) who stated that they had never been vaccinated against influenza also reporting having a bad reaction to a previous influenza vaccine. This suggests that some responses in our survey may represent reasons for non-vaccination in the previous influenza season rather than reasons for not ever having been vaccinated. Additionally, individuals who had been vaccinated in the past may be more likely to choose reasons other than “*Unnecessary*” and bias the estimate towards the null. However, given that the response to “Have you ever had a flu shot?” was neither the outcome nor explanatory variable, and the number of misclassified responses represented a relatively small proportion of the study sample, we believe this is unlikely to have had a significant effect on our estimates.

Second, CCHS respondents were permitted to select multiple reasons for not having been vaccinated against influenza, and we were unable to determine which was the primary reason. To address this uncertainty, we undertook additional analyses that incorporated a third outcome variable and determined that the overlap in selecting multiple reasons had no effect on the odds ratios calculated. Furthermore, the same association with self-rated health was observed in those who selected multiple reasons for non-vaccination, where at least one of these reasons fell under our classification of “*Unnecessary*”, suggesting that this belief was likely the primary factor driving an individual’s decision not to vaccinate. Conceptually this makes sense, many of the other possible CCHS responses are likely to vary from year to year, such as “*personal or family responsibilities*” or “*not available at time required*”, and may represent an individual’s reason for non-vaccination during the most recent influenza season; however, it is likely the belief that influenza vaccination is unnecessary that drive individuals never having been immunized against influenza.

Finally, we note that self-reported asthma is not a perfect proxy for a clinical diagnosis. The CCHS questions pertaining to self-reported asthma have not been tested for reliability or validity, although studies suggest there is good agreement between medical records and self-reported asthma, with a mean sensitivity of 68% (range 48–100%) and mean specificity of 94% (range 78–100%) when comparing self-reported asthma to a clinical diagnosis [28–30]. Two longitudinal studies assessing reliability indicate that recall of asthma is high but may be biased with respect to disease severity, with under-reporting from those with mild asthma [31,32]. Consequently, the respondents included in our study may not be fully representative of all asthma patients across Canada. However, bias would only be introduced into our study if there were correlation between likelihood of self-identification as asthmatic and mis-reporting of vaccination status. While possible we have no evidence to suggest that this is the case.

### Implications for clinical practice

Many individuals with chronic conditions attempt to “normalize” their condition, developing ways to manage and adapt to symptoms [33], which may explain the high proportion of adults with asthma who self-rated their health as good to excellent and who, in turn, perceive influenza vaccination as unnecessary. The Precaution Adoption Process Model described by Weinstein [34] provides a framework for understanding how we might change this perception. The model describes seven stages through which people progress when making a health-related decision, being unaware of an issue (stage 1), to aware but unengaged (stage 2), to acting on that issue (stage 6), to maintaining that action (stage 7). Here, our desired action is vaccination, with our respondents largely in stage 2 –aware of the importance of influenza vaccination but not yet seeing how the issue is relevant to them. Different factors stimulate the transition between each stage, with factors including personal experience with an issue, communications from significant others, recommendations from trusted individuals, and beliefs about personal susceptibility to illness and the effectiveness of the suggested intervention [35], motivating progress out of stage 2 towards deciding to vaccinate. Given that primary healthcare providers have a great deal of influence in the vaccination decisions of their patients [36], encouraging physicians to personalize the risks of non-vaccination to their patients with asthma, to recommend vaccination, and to provide information on the effectiveness of influenza vaccination in asthmatic adults might be the best strategy. Because nearly 16% of Canadians do not have a regular medical doctor [37], mass media-based awareness campaigns may also motivate the decision to act, and ultimately increase the rate of vaccination in this vulnerable group of individuals.

## Supporting information

S1 Table Binomial and multinomial logistic regression analyses of the relationship between self-rated health and the reasons for non-vaccination (*Unnecessary*–Yes *vs*. No and Yes(both)† *vs*. No), against seasonal influenza among Canadian adults with asthma, Canadian Community Health Survey pooled cycles 3.1(2005), 2007/08, 2009/10, 2011/12. (DOCX)

## References

[1] Webster RG, Monto AS, Braciale TJ, Lamb RA. Textbook of Influenza. Oxford (UK): John Wiley & Sons; 2014.

[2] World Health Organization. WHO | Influenza [Internet]. 25 Jan 2008 [cited 20 Apr 2016]. http://www.who.int/immunization/topics/influenza/en/

[3] Schanzer DL, Sevenhuysen C, Winchester B, Mersereau T. Estimating Influenza Deaths in Canada, 1992–2009. PLoS ONE. 2013;8: e80481 10.1371/journal.pone.0080481 24312225 PMC3842334

[4] Locksley RM, Fahy JV. Asthma and the flu: a tricky two-step. Immunol Cell Biol. 2014;92: 389–391. 10.1038/icb.2014.24 24687019 PMC4396068

[5] Schrag SJ, Shay DK, Gershman K, Thomas A, Craig AS, Schaffner W, et al Multistate Surveillance for Laboratory-Confirmed, Influenza-Associated Hospitalizations in Children: 2003–2004. Pediatr Infect Dis J. 2006;25: 395–400. 10.1097/01.inf.0000214988.81379.71 16645501

[6] Government of Canada SC. Asthma, by age group and sex (Number of persons) [Internet]. 17 Jun 2015 [cited 20 Apr 2016]. http://www.statcan.gc.ca/tables-tableaux/sum-som/l01/cst01/health49a-eng.htm

[7] Government of Canada PHA of C. Public Funding for Influenza Vaccination by Province/Territory (as of March 2014) [Internet]. 21 May 2015 [cited 20 Apr 2016]. http://www.phac-aspc.gc.ca/im/ptimprog-progimpt/fluvacc-eng.php#t1fn6

[8] Vozoris NT, Lougheed MD. Influenza vaccination among Canadians with chronic respiratory disease. Respir Med. 2009;103: 50–58. 10.1016/j.rmed.2008.08.004 18818066

[9] Wu S. Sickness and preventive medical behavior. J Health Econ. 2003;22: 675–689. 10.1016/S0167-6296(03)00042-0 12842321

[10] O’Halloran AC, Lu P, Williams WW, Bridges CB, Singleton JA. Influenza Vaccination Coverage Among People With High-Risk Conditions in the U.S. Am J Prev Med. 2016;50: e15–e26. 10.1016/j.amepre.2015.06.008 26238603 PMC4770254

[11] Nowak GJ, Sheedy K, Bursey K, Smith TM, Basket M. Promoting influenza vaccination: Insights from a qualitative meta-analysis of 14 years of influenza-related communications research by U.S. Centers for Disease Control and Prevention (CDC). Vaccine. 2015;33: 2741–2756. 10.1016/j.vaccine.2015.04.064 25936726 PMC5856146

[12] Lu P-J, Euler GL, Callahan DB. Influenza vaccination among adults with asthma findings from the 2007 BRFSS survey. Am J Prev Med. 2009;37: 109–115. 10.1016/j.amepre.2009.03.021 19589448

[13] Chi R-C, Reiber GE, Neuzil KM. Influenza and Pneumococcal Vaccination in Older Veterans: Results from the Behavioral Risk Factor Surveillance System. J Am Geriatr Soc. 2006;54: 217–223. 10.1111/j.1532-5415.2005.00577.x 16460371

[14] Chen Y, Yi Q-L, Wu J, Li F. Chronic disease status, self-perceived health and hospital admissions are important predictors for having a flu shot in Canada. Vaccine. 2007;25: 7436–7440. 10.1016/j.vaccine.2007.08.003 17825962

[15] Santos-Sancho JM, Andrés AL, Jimenez-Trujillo I, Hernández-Barrera V, Carrasco-Garrido P, Astasio-Arbiza P, et al Adherence and factors associated with influenza vaccination among subjects with asthma in Spain. Infection. 2013;41: 465–471. 10.1007/s15010-013-0414-2 23404684

[16] Government of Canada SC. Public Use Microdata File (PUMF) Collection [Internet]. 17 Jan 2011 [cited 4 Feb 2017]. http://www5.statcan.gc.ca/olc-cel/olc.action?objId=11-625-X&objType=2&lang=en&limit=0

[17] Statistics Canada. Trends in influenza vaccination in Canada, 1996/1997 to 2005: Findings [Internet]. [cited 20 Apr 2016]. http://www.statcan.gc.ca/pub/82-003-x/2006010/article/vaccination/4060740-eng.htm

[18] Dubé E, Gagnon D, Kiely M, Defay F, Guay M, Boulianne N, et al Seasonal influenza vaccination uptake in Quebec, Canada, 2 years after the influenza A(H1N1) pandemic. Am J Infect Control. 2014;42: e55–e59. 10.1016/j.ajic.2014.01.006 24773805

[19] Thomas S, Wannell B. Combining cycles of the Canadian Community Health Survey. Health Rep. 2009;20: 53–8. 19388369

[20] Government of Canada SC. Canadian Community Health Survey—Annual Component (CCHS) [Internet]. 1 Jan 2015 [cited 20 Apr 2016]. http://www23.statcan.gc.ca/imdb/p2SV.pl?Function=getSurvey&SDDS=3226&lang=en&db=imdb&adm=8&dis=2

[21] Manor O, Matthews S, Power C. Self-rated health and limiting longstanding illness: inter-relationships with morbidity in early adulthood. Int J Epidemiol. 2001;30: 600–607. 11416091 10.1093/ije/30.3.600

[22] The University of British Columbia, Board of Governors. Research Involving Human Participants. Policy No. 89 [Internet]. Jun 2012 [cited 20 Apr 2016]. http://universitycounsel.ubc.ca/files/2012/06/policy89.pdf

[23] CDC—Asthma—Most Recent Asthma Data [Internet]. [cited 22 Apr 2016]. http://www.cdc.gov/asthma/most_recent_data.htm

[24] Centers for Disease Control and Prevention. Flu and People with Asthma| Seasonal Influenza (Flu) | CDC [Internet]. 14 Aug 2015 [cited 20 Apr 2016]. http://www.cdc.gov/flu/asthma/index.htm

[25] Kam K, McConnell A. Influenza vaccination among household contacts of children with cystic fibrosis and healthy children. Paediatr Child Health. 2013;18: e55–e58. 24426812 PMC3885107

[26] Zimmerman RK, Raymund M, Janosky JE, Nowalk MP, Fine MJ. Sensitivity and specificity of patient self-report of influenza and pneumococcal polysaccharide vaccinations among elderly outpatients in diverse patient care strata. Vaccine. 2003;21: 1486–1491. 12615445 10.1016/s0264-410x(02)00700-4

[27] Mac Donald R, Baken L, Nelson A, Nichol KL. Validation of self-report of influenza and pneumococcal vaccination status in elderly outpatients. Am J Prev Med. 1999;16: 173–177. 10198654 10.1016/s0749-3797(98)00159-7

[28] Torén K, Brisman J, Järvholm B. Asthma and asthma-like symptoms in adults assessed by questionnaires. A literature review. Chest. 1993;104: 600–608. 7802735 10.1378/chest.104.2.600

[29] Linet MS, Harlow SD, McLaughlin JK, McCaffrey LD. A comparison of interview data and medical records for previous medical conditions and surgery. J Clin Epidemiol. 1989;42: 1207–1213. 2585011 10.1016/0895-4356(89)90119-4

[30] Harlow SD, Linet MS. Agreement between questionnaire data and medical records. The evidence for accuracy of recall. Am J Epidemiol. 1989;129: 233–248. 2643301 10.1093/oxfordjournals.aje.a115129

[31] Mirabelli MC, Beavers SF, Flanders WD, Chatterjee AB. Reliability in reporting asthma history and age at asthma onset. J Asthma. 2014;51: 956–963. 10.3109/02770903.2014.930480 24894742 PMC4514017

[32] Torén K, Palmqvist M, Löwhagen O, Balder B, Tunsäter A. Self-reported asthma was biased in relation to disease severity while reported year of asthma onset was accurate. J Clin Epidemiol. 2006;59: 90–93. 10.1016/j.jclinepi.2005.03.019 16360566

[33] Robinson CA. Managing Life with a Chronic Condition: The Story of Normalization. Qual Health Res. 1993;3: 6–28. 8457793 10.1177/104973239300300102

[34] Weinstein ND. The precaution adoption process. Health Psychol Off J Div Health Psychol Am Psychol Assoc. 1988;7: 355–386. 10.1037//0278-6133.7.4.355 3049068

[35] “The Precaution Adoption Process Model” by Weinstein ND, Sandman PM, Blalock SJ, in Glanz K, Rimer BK, Viswanath K. Health behavior and health education theory, research, and practice. San Francisco, CA: Jossey-Bass; 2008.

[36] Villacorta R, Sood N. Determinants of healthcare provider recommendations for influenza vaccinations. Prev Med Rep. 2015;2: 355–370. 10.1016/j.pmedr.2015.04.017 26844092 PMC4721324

[37] Government of Canada SC. Access to a regular medical doctor, 2013 [Internet]. 12 Jun 2014 [cited 20 Apr 2016]. http://www.statcan.gc.ca/pub/82-625-x/2014001/article/14013-eng.htm

