# Supplementary material for: Self-rated health and reasons for non-vaccination against seasonal influenza in Canadian adults with asthma
Source: PLoS One. 2017 Feb 16;12(2):e0172117. doi: 10.1371/journal.pone.0172117 (PMC5312957; doi:10.1371/journal.pone.0172117)
Supplement: S1 Table — (DOCX) [file pone.0172117.s001.docx]

| **Table S1.** Binomial and multinomial logistic regression analyses of the relationship between self-rated health and the reasons for non-vaccination (*Unnecessary* – Yes *vs.* No and Yes(both)† *vs.* No), against seasonal influenza among Canadian adults with asthma, Canadian Community Health Survey pooled cycles 3.1(2005), 2007/08, 2009/10, 2011/12. | | | | |
| --- | --- | --- | --- | --- |
|  | **Binomial Model** | **Multinomial Model** | | |
|  | **Yes† *vs* No**  **OR (95% CI)** | **Yes† *vs* No**  **OR (95% CI)** | **Yes(both)‡ *vs* No**  **OR (95% CI)** | |
| **Self-rated Health** |  |  |  | |
| Poor | Reference | Reference | Reference | |
| Fair | 1.42 (0.97, 2.09) | 1.42 (0.96, 2.11) | 1.38 (0.61, 3.16) | |
| Good | 1.67 (1.19, 2.34) | 1.68 (1.19, 2.37) | 1.34 (0.65, 2.76) | |
| Very Good | 2.60 (1.84, 3.67) | 2.60 (1.83, 3.69) | 2.55 (1.23, 5.27) | |
| Excellent | 2.64 (1.74, 3.99) | 2.66 (1.75, 4.05) | 2.11 (0.93, 4.82) | |
| **Adjusted for sex, age, province/territory and education*  *† Yes represents individuals that selected only a perceptual reason*  *‡Yes(both) represents individuals that selected both a perceptual(unneccessary) and technical reason* | | | |  |
